# Hepatic stellate cells: central modulators of hepatic carcinogenesis

**DOI:** 10.1186/s12876-015-0291-5

**Published:** 2015-05-27

**Authors:** Alexandra I Thompson, Kylie P Conroy, Neil C Henderson

**Affiliations:** MRC Centre for Inflammation Research, The Queen’s Medical Research Institute, University of Edinburgh, 47 Little France Crescent, Edinburgh, UK

## Abstract

Hepatocellular carcinoma (HCC) represents the second most common cause of cancer-related death worldwide, and is increasing in incidence. Currently, our therapeutic repertoire for the treatment of HCC is severely limited, and therefore effective new therapies are urgently required. Recently, there has been increasing interest focusing on the cellular and molecular interactions between cancer cells and their microenvironment. HCC represents a unique opportunity to study the relationship between a diseased stroma and promotion of carcinogenesis, as 90 % of HCCs arise in a cirrhotic liver. Hepatic stellate cells (HSC) are the major source of extracellular proteins during fibrogenesis, and may directly, or via secreted products, contribute to tumour initiation and progression. In this review we explore the complex cellular and molecular interplay between HSC biology and hepatocarcinogenesis. We focus on the molecular mechanisms by which HSC modulate HCC growth, immune cell evasion and angiogenesis. This is followed by a discussion of recent progress in the field in understanding the mechanistic crosstalk between HSC and HCC, and the pathways that are potentially amenable to therapeutic intervention. Furthermore, we summarise the exciting recent developments in strategies to target HSC specifically, and novel techniques to deliver pharmaceutical agents directly to HSC, potentially allowing tailored, cell-specific therapy for HCC.

## Introduction

Hepatocellular carcinoma (HCC) represents the second most common cause of death from cancer worldwide, and was responsible for nearly 746 000 deaths in 2012 [1–3]. In patients with cirrhosis, HCC is the most common cause of death. Worldwide, chronic hepatitis B virus infection remains the major risk factor, with 80 % of cases occurring in eastern Asia and sub-Saharan Africa. In most countries, the mortality rate of HCC approximates the incidence, which is increasing [4–6]. This is partly due to the rising prevalence of advanced fatty liver disease and chronic hepatitis C, alongside other risk factors such as hepatitis B infection and alcohol-related cirrhosis. Some progress has been made with prevention, for example emerging antiviral agents and vaccination for hepatitis B. However, the vast majority of HCC cases are associated with fibrosis, and 90 % of tumours develop in cirrhotic livers [4, 5, 7–10]. Furthermore, liver disease severity markers correlate with tumour formation [4–6, 9, 11–14]. Currently there are no effective anti-fibrotic therapies available to halt the fibrosis-cirrhosis-HCC continuum. Patients who present with early disease may benefit from resection, transplantation or loco-regional therapy, however many are unsuitable for curative treatment due to advanced malignancy, or the severity of co-existing liver disease. The multi-tyrosine kinase inhibitor sorafenib is the only available systemic chemotherapy agent with survival benefit for advanced stage HCC, however its use is limited to those with well-preserved liver function [11]. Whilst there is scope to optimize our use of existing treatments, for example by targeting tumours earlier and combining local and systemic approaches, efforts to broaden our chemotherapy armamentarium have been disappointing. Numerous molecular therapies with robust preclinical evidence for efficacy have failed to show benefit in clinical trials. This may in part reflect the abnormal tumour microenvironment, which acts to support the persistence and growth of cancer cells, and has resulted in the peri-tumoural stroma and its cellular inhabitants becoming an intense area of study in the search for efficacious therapies for HCC.

In this review we focus on the complex interplay between hepatic stellate cell (HSC) biology and hepatocarcinogenesis. The mechanisms by which HSC may facilitate HCC development and progression are likely to involve diverse biological processes including regulation of extracellular matrix (ECM) turnover, growth factor and cytokine signalling, promotion of tumour angiogenesis and immunomodulation. We will discuss how this burgeoning area of research may yield exciting new therapies for patients with HCC.

### Role of the stroma in hepatocarcinogenesis

The stroma is a central component of both hepatic fibrosis and carcinogenesis, and is a key player in the cellular and molecular mechanisms linking these processes. It is still unclear, however, whether liver fibrosis specifically promotes HCC, or if it is merely a wound-healing by-product of chronic hepatic injury and inflammation, with no direct impact on liver cancer formation [8, 13–15]. Evidence would suggest the former; the identification of gene signatures from non-tumoural tissue correlating with late recurrence of HCC, supports the concept of a ‘field effect’ in cancer development [9, 11, 13, 14, 16–25].

Following liver injury, quiescent HSC become activated to matrix-secreting myofibroblasts and are the major source of ECM proteins during liver fibrogenesis [8, 13, 26]. As master regulators of the fibrotic matrix, HSC may therefore directly influence HCC formation via effects on the tumour stroma. Furthermore, it is well established in other systems that complex intercellular signalling networks exist between tumours and cancer-associated fibroblasts, contributing to cancer initiation, growth and progression [8, 13, 16–19, 21–26]. Tumour secretion of cytokines such as transforming growth factor-β (TGF-β), stimulate myofibroblast activation leading to profound changes in ECM composition and organization. Therefore, HSC or HSC-secreted products may be either permissive or necessary for oncogenesis and HCC persistence. In other cancers, the identification of pathways that the tumour depends upon for growth and proliferation, so-called “oncogenic addiction loops” has led to revolutionary therapeutic approaches. The landmark discovery of the protein kinase oncogene BCR-ABL and subsequent development of imatinib, allowed curative treatment of chronic myeloid leukaemia, and has paved the way for targeted therapies in other malignancies [27, 28]. Despite extensive genomic profiling of HCC, targeting other non-kinase oncogenes such as RAS and MYC has proven more challenging. The identification of promising candidate pathways targeting inhibition of a driving molecular alteration, which is also applicable in a significant proportion of patients, remains an elusive yet alluring goal [29]. Furthermore, the microenvironment may modulate susceptibility to inhibition of specific oncogenic pathways. Straussman *et al.* developed a co-culture system to test the ability of 23 stromal cell types to influence the susceptibility of 45 different cancer cell lines to 35 therapeutic agents [7]. They demonstrated that stroma-mediated resistance to anti-cancer drugs (especially targeted agents) is common. In particular, although melanomas expressing mutant BRAF respond to vemurafenib, hepatocyte growth factor (HGF) secretion by peri-tumoural stromal cells correlated with resistance to vemurafenib-induced cell death [7, 30, 31]. This illustrates the importance of stroma-derived resistance to chemotherapy, in many different organs and disease settings. Therefore, in the search for key driver mutations in HCC, the effect of the microenvironment cannot be underestimated. This may necessitate combinations of chemotherapeutic agents, to neutralize specific stromal interactions, resulting in greater overall clinical efficacy.

### HSC in HCC

It is well-known that activated HSC infiltrate HCC stroma and peri-tumoural tissue, and are localised around tumour sinusoids, fibrous septae and the tumour capsule [32–34]. Activated HSC have also been identified around the periphery of dysplastic nodules within the liver [35]. Following activation to the myofibroblast phenotype, HSC secrete substantial amounts of ECM proteins into the stroma. Fibrotic matrix deposition and degradation by HSC is tightly regulated in the liver. For example, tissue inhibitors of metalloproteinases 1 (TIMP-1) secretion favours scar deposition by inhibiting the endogenous matrix-degrading activities of various matrix metalloproteinases (MMPs). However, the balance of TIMPs and MMPs is complex; activated HSC are also a major source of MMP-2 *in vitro*, elevation of which has been correlated with increased tumoural collagen I, extracellular remodeling, and HCC progression [12, 36, 37]. Interestingly, the biomechanics of the ECM are also relevant. Differentiation of primary hepatocytes is inhibited by culture on a stiff collagen gel, with accompanying promotion of proliferation [38, 39]. *In vitro* increasing matrix stiffness has also been shown to directly stimulate growth of the HCC cell lines, HuH-7 and HepG2, and reduce chemotherapy-induced apoptosis [40]. Integrin β1 signalling was an integral driver of this response, via Fak, Erk, Pkb/Akt and Stat3 pathways [40]. Furthermore, stromal stiffness is self-perpetuating, causing stellate cell activation, and therefore further fibrosis [15, 41, 42]. Data in humans support these experimental findings. Ultrasound elastography has demonstrated that measurements of liver stiffness predict HCC development [43–46]. Similarly, established HCC demonstrates further increases in matrix stiffness, more so than the peri-tumoural hepatic parenchyma [47]. The mechanical tension provided by an altered ECM is likely to act on HCC development and progression via outside-in signalling, for example by integrins, (discussed below) to support tumour growth and progression. This has also been observed in other malignancies, such as a mouse model of breast cancer [48]. Hepatocarcinogenesis in the context of cirrhosis, however, is a unique model of diseased ECM, and an ideal setting to further characterise and potentially target stromal drivers.

### Integrins as mediators of HSC/HCC crosstalk

Consisting of an α- and β-subunit, integrins form a family of transmembrane receptors that ‘integrate’ the extracellular and intracellular environments through binding ECM and the cytoskeleton [49]. Via transduction of signals between the internal and external cellular domains, integrins regulate cell adhesion, spreading, migration, proliferation and differentiation as well as ECM deposition and remodelling [50].

In activated HSC downstream integrin signalling, via the focal adhesion kinase (FAK)-phosphatidylinositol 3-kinase (PI3K)-Akt signaling pathway, promotes ECM deposition [51]. Increased ECM stiffness *in vitro* enhances integrin expression and activity and focal adhesion formation, [48] with subsequent activation of downstream integrin signalling within the hepatocyte that may nurture the growth and survival of precancerous cells. Matrix stiffness has been reported to dictate differentiation and chemotherapeutic resistance of human HCC cell lines, with softer matrices abrogating hepatoma proliferation and stiffer platforms promoting proliferation [40, 52]. In an elegant *in vivo* study, cells from the HCC cell line McA-RH7777 were implanted into rats treated with carbon tetrachloride (CCl_4_) for varying lengths of time, thereby modelling tumourigenesis on different liver stiffness backgrounds. Microarray analysis of the tumours demonstrated a positive correlation between matrix rigidity and tumour angiogenesis [52]. Correlations between collagen expression, integrin expression and tumourigenicity have also been reported in human HCC and murine HCC models [53, 54]. Characterisation of integrin expression in hepatoma cell lines has revealed a high degree of heterogeneity in integrin expression [55]. Comparing two clinically relevant mouse models of HCC, platelet-derived growth factor (PDGF)-C overexpressing and PTEN null mice, Lai *et al.* demonstrated that each model had a specific pattern of integrin gene expression, further indicating HCC heterogeneity [54].

The β1 integrin subfamily has been extensively studied in the context of HCC, and hepatocarcinogenesis is associated with the enhanced expression of integrins α1β1, α2β1 and α3β1 and the acquisition of a migratory phenotype by hepatocytes [56–58]. Further, assessment of integrin β1 expression in human HCC tissues demonstrated a positive correlation with ECM stiffness, pathological grade and metastasis [59]. Blockade of integrin β1 *in vitro* significantly abrogates migration and invasion of HCC cell lines induced by TGF-β1 and epidermal growth factor (EGF) [58, 60]. Conversely, overexpression of integrin β1 has been reported to enhance HepG2 cell migration [61]. More recently it has been reported that integrin β1 is involved in the transduction of ECM signalling into HCC cells, resulting in the downstream activation of angiogenic signalling [52]. Utilising a high-stiffness gel to culture HCC cell lines Dong *et al.* found that vascular endothelial growth factor (VEGF) expression is suppressed by treatment with an integrin β1-specific antibody [52]. SERPINA5 (Protein C inhibitor), a member of the serine protease inhibitor superfamily know to have anti-metastatic and anti-angiogenic effects, [62] is down-regulated in human HCC tissues and further assessment of it’s anti-tumourigenic activity demonstrated that this was mediated by effects on the fibronectin-integrin β1 signalling pathway [63]. The relationship between integrin β1 and ECM stiffness in HCC is further highlighted in a study where resistance of the HCC cell line, Hep3B, to sorafenib was found to be mediated by integrin β1 and its downstream effector JNK [64].

Other integrin subunits, in addition to β1, have been reported to have key roles in HCC progression. Fan *et al.* have reported integrin α6 expression to strongly correlate with HCC metastasis in humans [65]. Integrin α6 overexpression in HCC cell lines (utilising a viral short hairpin RNA-mediated strategy) revealed that integrin α6 can form a complex with CD151, a tetraspanin protein also associated with HCC invasion [65]. Further investigation *in vivo* indicates that the CD151/α6 complex stimulates the PI3K-Akt signalling pathway leading to enhanced epithelial-mesenchymal-transition (EMT) of HCC cell lines [65].

Crosstalk between integrins and TGF-β signalling has also been studied in hepatocarcinogenesis. TGF-β receptor I (TGF-β RI) activation has been reported to promote HCC cell invasiveness through phosphorylation of the intracellular portion of the β1 subunit of the α5β1 integrin via Smad-2 and Smad-3, leading to an inside-out conformational change and stimulating vascular invasion [66]. Up-regulation of other integrins including α3β1 and α6β1 by TGF-β1 has also been reported, leading to increased tumour invasiveness into surrounding tissues [67]. Furthermore specific crosstalk between fibronectin-binding integrins and TGF-β1 can promote cell cycle progression in HCC cells through activation of c-Src [68]. Crosstalk between integrins, growth factor receptors and ECM proteins including collagen, have further been shown to alter downstream signal transduction pathways such as Smad, promoting both hepatocyte proliferation and sustaining HSC activation [69, 70]. TGF-β1 has also been reported to modulate α5β1 expression and synergistically enhance integrin-mediated FAK phosphorylation and cell adhesion in the HCC cell line SMMC-7721 [71]. Therefore, integrins (via modulation of TGF-β signalling) may render hepatocytes less sensitive to pro-apoptotic signals in early HCC stages, and more sensitive to tumourigenic differentiation and metastasis formation in advanced HCC.

### HSC growth factor signalling

HSC have been shown to favour HCC tumourigenicity, potentially as a result of a change in their secretory phenotype upon activation. *In vitro* studies, using conditioned media from activated HSC, have consistently reported increased proliferation, migration and invasion of tumour cells [72–74]. Isolation and subsequent co-culture of human intratumoural HSC with hepatoma cell lines enhanced their viability and migratory capacity [72]. Furthermore, co-transplantation with HCC cells into nude mice promoted tumour formation and growth [75]. Utilising both co-culture and conditioned media from primary human HSC Giannelli and colleagues determined Laminin-5 to be a mediator of HSC-induced HCC migration via its activation of the MEK/ERK pathway [76]. This is supported by *in vivo* experiments, in which co-transplantation of murine activated HSC with murine HCC cells (H22 line) into immunocompetent mice resulted in significantly larger tumour volumes [73]. Furthermore, implantation of human HCC cell lines (PLC and Hep3B) into nude mice did not form tumours unless activated HSC were concurrently implanted [72]. HepG2 cells did form tumours when implanted alone, however tumour growth was more rapid when co-transplanted with activated HSC [72]. Activated HSC secrete a broad range of growth factors including HGF, TGF-β, fibroblast growth factor (FGF), EGF, VEGF and insulin-like growth factor (IGF). The following sections discuss how these growth factors are involved in HCC pathogenesis.

### Hepatocyte Growth Factor

HGF is expressed by HSC and myofibroblasts, [77, 78] and is a highly potent hepatocyte growth factor regulating cell proliferation, migration, survival and angiogenesis [79–82]. As such it is widely regarded as a key factor for tumour cell invasion and metastasis [83]. HGF binding to its receptor, c-MET, induces receptor homodimerization and a subsequent phosphorylation cascade. A transmembrane receptor tyrosine kinase, c-MET is found in 20-48 % of HCCs, [84–86] and has been shown to be expressed by multiple HCC cell lines [72]. Correlations between increased c-MET and HCC tumour size or invasiveness of HCC have been reported in some studies [87, 88]. c-MET overexpression is also associated with a reduced five-year HCC survival, and a c-MET-regulated expression signature has been reported to define a subset of patients with poor prognosis and an aggressive phenotype [89, 90]. Within HCC tumours, activated HSC have been found to initiate signalling pathways downstream of c-MET, including NF-κB and ERK leading to tumour proliferation and migration [72, 91].

The pro-tumourigenic activity of fibroblast-secreted HGF has also been reported *in vitro.* Conditioned media from isolated and activated HSC, pre-incubated with anti-HGF antibodies, was found to abrogate the proliferative and migration-inducing effects on HCC cell lines, seen in non-treated conditioned media [72]. This has also been demonstrated in cancer-associated fibroblasts (CAF) isolated from HCC, where treatment of CAF-conditioned media with an anti-HGF antibody significantly reduced HCC proliferation in Hep3B and MHCC97L cell lines [74]. Moreover, a HGF/c-MET specific antagonist, NK4, has been found to inhibit markedly the fibroblast-induced invasion of cancer cells, both *in vitro* and *in vivo,* [92–94] although this has yet to be translated into the clinical setting. A murine model of HCC with similarities to the human disease was recently developed, in which progressive fibrosis and cirrhosis, initiated by ectopic expression of PDGF-C, precedes hepatocyte dysplasia and eventual HCC development [95]. Analysis of these PDGF-C transgenic mice demonstrated that expression of hepatic HGF and its receptor were elevated at the time point at which dysplastic foci are present, further suggesting a pro-tumourigenic role for HGF. Activation of HGF/c-MET signalling has also been shown to enhance HCC chemoresistance. Conditioned media from the activated HSC cell line LX-2 enhanced resistance of the HCC cell line Hep3B to the chemotherapeutic agent cisplatin, an effect mediated by HGF [96]. Tumour cells may also potentiate pro-metastatic c-MET signalling via an autocrine mechanism involving TIMP-1, leading to downstream expression of metastasis-promoting genes [97, 98].

However, HGF signalling is not unidirectional. A high level of bi-directional crosstalk between tumour cells and stromal cells, in particular fibroblasts, has been reported. Nakamura and colleagues have reported the expression of HGF inducers in several carcinoma cell lines, including squamous cell carcinoma, human epidermoid carcinoma, human non-small cell lung cancer cells, human cholangiocarcinoma cells, and SBC-3 human small cell lung carcinoma cells [99]. These HGF inducers include interleukin (IL)-1β, FGF, PDGF and TGF-α and were reported to up-regulate HGF expression by stromal fibroblasts [99, 100]. Taken together, these studies highlight that HGF and aberrant c-MET signalling have a critical role in mediating the bi-directional crosstalk between HSC and tumour cells during hepatocarcinogenesis.

### Transforming growth factor-β

The large latent TGF-β complex is secreted by most cell types, including human HSC and hepatocytes [101, 102] and fixed in the ECM by transglutaminase-dependent linkage of latent TGF-β binding protein to fibronectin and other ECM proteins, forming a reservoir of latent TGF-β. In the context of HCC, it has been suggested that defective TGF-β signalling promotes tumourigenesis secondary to reduced responsiveness to the anti-proliferative effects of TGF-β signalling [103, 104]. However, TGF-β appears to exhibit multiple roles in HCC pathogenesis. Tumour-suppressor functions are observed in the early stages of liver damage and regeneration, whereas during cancer progression, TGF-β may exacerbate tumour invasiveness and metastatic behavior [105]. It has further been demonstrated that TGF-β and PDGF signaling crosstalk supports EMT and is crucial for tumour growth and the acquisition of an invasive phenotype [106].

The survival and malignancy of HCC cell lines, including Huh7 and HepG2, have been reported to require autocrine TGF-β signalling, with exogenous TGF-β leading to growth inhibition of HCC cells [107]. Utilising HCC cell lines, Meindl-Beinker *et al.* revealed a heterogeneic response to TGF-β, reflective of different stages and mechanisms of disease. Variation between cell lines in their endogenous TGF-β and Smad7 levels, and their transcriptional activity of Smad3, was related to the maintenance of TGF-β cytostatic activity. In particular, the Hep3B, HepG2 and PLC hepatoma cell lines were found to have low TGF-β and Smad7 levels and strong Smad3 transcriptional activity and were thus sensitive to TGF-β cytostatic activity, representative of the early stages of chronic liver disease [108]. In an analysis of TGF-β gene expression in HCC patients, Coulouarn *et al.* reported that those tumours displaying an invasive phenotype and increased recurrence were characterized by a late TGF-β signalling signature, with transcriptional activation of genes associated with matrix remodelling and cell adhesion [109].

Therefore, as the role of TGF-β in HCC pathogenesis appears to be highly context-dependent, exhibiting both pro- and anti-tumoural activity, it is highly unlikely that pan-TGF-β blockade will provide a useful therapeutic avenue in HCC treatment. More selective strategies to interfere with TGF-β signalling, perhaps even at a cell-specific level, will likely be required to modulate this signalling pathway for therapeutic gain in the context of HCC.

### Epiregulin

The gut microbiome is increasingly recognized as a powerful modulator of fibrosis, cirrhosis, and infectious complications in chronic liver disease. Much interest is currently focused on the translocation of bacterial pathogen-associated molecular patterns (PAMPs), which activate inflammatory responses through Toll-like receptors (TLRs). Recently Dapito *et al*. demonstrated that Tlr4*mut* mice (harbouring non-functional TLR4) that received diethylnitrosamine (DEN) and CCl_4_ show 80-90 % reduction in HCC tumour size and number, compared with mice expressing wild-type TLR4 [110]. Gut sterilisation significantly reduced this effect whereas LPS treatment enhanced it, suggesting a role for the LPS-TLR4 pathway in promotion of hepatocarcinogenesis. Interestingly, alongside hepatocytes, HSC were identified as candidates for TLR4-dependent tumour promotion in the chronically injured liver. LPS and the gut microbiome were found to induce HSC activation, resulting in production of the mitogens HGF and epiregulin, which likely act on malignant hepatocytes. Epiregulin is a member of the EGF family, and results in EGF receptor and human epidermal growth factor receptor 2 activation during early stages of DEN/CCl_4_ carcinogenesis, whereas it reduces hepatocyte apoptosis by NF-KB nuclear translocation during later stages [110, 111]. This suggests that there may be merit in evaluating whether long-term antibiotic treatment confers any protection against HCC development. This could initially be investigated by following up patients with cirrhosis on long-term prophylaxis for spontaneous bacterial peritonitis or encephalopathy, although identifying a comparable control group may prove challenging.

### HSC and angiogenesis

Angiogenesis has a critical role in HCC initiation, progression and metastasis, as reflected by the efficacy of sorafenib, which targets this process. The rapid growth pattern of malignant hepatocytes requires new vessel formation, stimulated by multiple pro-angiogenic factors. This pro-angiogenic environment in turn supports tumour progression and metastasis. The relevance of tumour vascularity is reinforced by the observation that VEGF expression progressively increases from low-grade dysplasia to early-stage HCC [112]. VEGF overexpression is also associated with high tumour grade, and vascular and portal vein invasion [113–117]. Furthermore, raised plasma VEGF and angiopoietin 2 (Ang-2) are independent predictors of poor prognosis in advanced HCC [118].

HSC are known to secrete VEGF as well as other angiogenic factors including PDGF, MMPs, FGF, TGF-β1, EGF, angiopoietin-1 (Ang-1) and Ang-2 [119–121]. Upon activation, HSC express multiple smooth muscle cell markers, suggesting they may act like pericytes during angiogenesis [122, 123]. They also express angiogenic growth factor receptors, such as VEGF receptor, PDGF receptor and Tie-2 [124–126]. In liver injury and HCC, this facilitates reciprocal signalling between HSC and endothelial cells or malignant hepatocytes and contributes towards a pro-angiogenic microenvironment. VEGF secretion by HSC can be hormonally induced by leptin, or by physical stress such as hypoxia, and is upregulated in HCC [120, 124, 127]. VEGF receptor upregulation also occurs during HSC activation, resulting in increased mitogenesis in response to VEGF [13].

Conditioned media from HCC cells can activate HSC and stimulate VEGF production. Coulouarn *et al.* co-cultured LX2 cells with HepRG HCC cells, and analysis of differential gene expression identified a gene network linked to VEGFA and MMP9 [128]. This was shown to promote angiogenesis, as conditioned medium from LX2-HepaRG coculture (but not LX2 or HepaRG medium alone) induced tubule complex formation by primary human umbilical vein endothelial cells. A gene signature of this cross-talk correlated with poor prognosis and metastasis in humans [128].

Lin *et al.* have also shown increased angiogenesis by activated HSC *in vitro* using a murine HCC cell line (H22) and rat colon microvascular endothelial cells [129]. They went on to demonstrate *in vivo*, using an orthotopic HCC model, that activated HSCs promote tumour vascularisation via increased VEGF and possibly PDGF secretion.

Of particular interest in HCC is the interaction between malignant hepatocytes, endothelial cells and activated HSC. Torimura *et al.* characterised expression of Ang-1, Ang-2 and Tie2 receptors in HCC cell lines (HLE and HuH-7) and human HCC cases [130]. They concluded angiopoietin-Tie2 signalling in the vascular wall may act in favour of vessel remodelling in HCC. Ang-2 production by hepatoma cells, HSC and smooth muscle cells binds Tie2 (on HSC, smooth muscle and endothelial cells) and destabilises connections between endothelial cells, perivascular support cells and ECM. This allows exposure to VEGF, which in these relatively hypoxic conditions, is upregulated. Proliferation of endothelial cells ensues, allowing neovascularization and further tumour growth.

Recently, it has been shown that metformin inhibits angiogenesis *in vitro*, in an HCC (HepG2 line) and HSC (LX2) co-culture system [131]. This was associated with reduced VEGF production. It was postulated that metformin was acting via AMPK activation, and specifically targeting HSC in this model. Indeed, inhibition of AMPK on LX2 cells (but not on HepG2 cells) using siRNA did restore VEGF levels and abrogate metformin’s anti-angiogenic effect. Metformin would seem a promising candidate for human HCC treatment, but unfortunately retrospective data would suggest a lack of survival benefit [132]. However, considering the well-established tolerability of metformin, its potential HSC-mediated effect on angiogenesis merits further investigation.

**Fig. 1:**
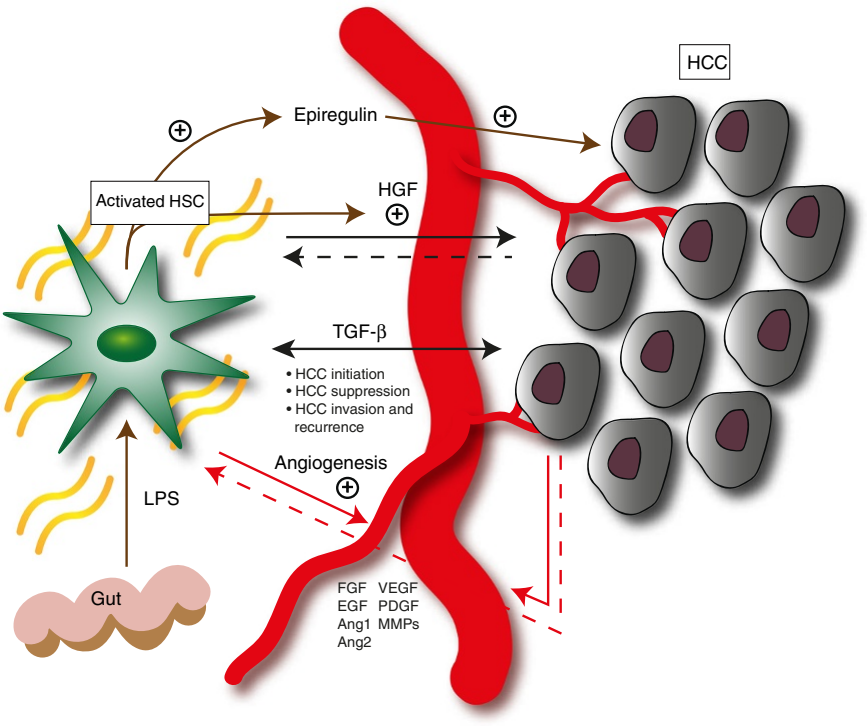
Crosstalk between HSC and HCC. HSC-secreted factors such as HGF may promote hepatocarcinogenesis. Similarly, HCC signalling results in further HGF production from activated HSC. TGF-β demonstrates both tumour-suppressive and tumour-promoting functions, depending on context. HSC produce angiogenic cytokines, supporting new vessel growth. HCC cells contribute to angiogenic signalling, and HSC also possess receptors for some of these factors. Gut-derived LPS induces HSC activation, resulting in epiregulin and HGF production, with mitogenic effects on HCC

Some of the factors mediating crosstalk between HSC and HCC are summarised in Fig. 1.

### HSC and immunomodulation

Tumour immune evasion is now regarded as a hallmark of cancer progression and is therefore a very active area of research. One mechanism by which tumours evade the immune response is through the augmentation of the numbers and activity of immunosuppressive cells, at both the tumour site and within lymphoid organs [133]. Such cells include regulatory T-cells (Tregs) and myeloid-derived suppressor cells (MDSC). Increased levels of Tregs within peripheral blood and tumours have been reported in human HCC cases, and have further been shown to suppress anti-tumour immune responses in addition to promoting angiogenic remodeling [134–136]. Further, intratumoural Treg accumulation has been reported to correlate with disease progression and poor prognosis [137]. MDSC are defined by the markers CD11b and Ly6-C/G and have been found in the tumour, lymph nodes and blood, suppressing cellular responses to cancer cells [138].

The immunosuppressive activities of HSC have only recently been recognised with studies demonstrating, both *in vitro* and *in vivo*, that activated HSC are able to strongly suppress T-cell responses. Investigation into the divergent immunomodulatory activity of quiescent and intratumoural HSC has revealed that, *in vitro*, intratumoural HSC induce T-cell hyporesponsiveness, an effect not seen with quiescent HSC [139]. Moreover, in an orthotopic rat model of HCC, intratumoural HSC number strongly correlated with T-cell apoptosis and lung metastatic nodules [140]. Although a direct interaction was not reported, this does suggest an additional role for HSC in HCC metastasis via an immunosuppressive mechanism.

Co-transplantation of HCC cells and HSC into immunocompetent mice promoted HCC proliferation and enhanced tumour angiogenesis, in association with inhibition of lymphocyte infiltration and apoptosis of infiltrating monocytes [73]. In an orthotopic model of HCC, activated HSC in tumour-bearing mice significantly increase Treg and MDSC populations in the spleen and tumour stroma [141]. An increase in tumour vascular and lymphatic vessel density was also reported in those tumours co-transplanted with HSC.

Investigation into the mechanisms underlying HSC immunomodulatory effects in HCC has demonstrated that this may be mediated via upregulation of human B7 homolog 1 (B7-H1; programmed death ligand 1 (PDL-1)) on tumoural HSC [142–144]. B7-H1 can act as both receptor and ligand and has immunosuppressive functions such as promoting activated T-cell apoptosis and inhibiting T-cell-mediated tumour cell apoptosis [1, 145, 146]. Its counter-receptor, PD-1, is expressed on activated, but not resting, T-cells, B-cells and monocytes [2]. B7-H1/PD-1 signaling has been reported to promote Treg cell induction and immunosuppressive function through the down-regulation of mTOR and AKT phosphorylation [147, 148]. *In vitro* experiments involving incubation of T-cells with anti-B7-H1 monoclonal antibody resulted in a significant reduction in HSC immunomodulatory activity and HCC migration and invasion [139].

**Fig. 2:**
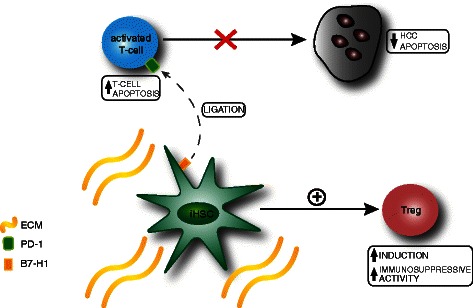
Immunomodulatory effects of HSC in HCC. Intratumoural HSC (iHSC) promote HCC progression through i) an increase in Treg cell induction and immunosuppressive function and ii) upregulation of B7-H1 on iHSC resulting in increased ligation of its receptor (PD-1) on activated T-cells, leading to increased apoptosis of activated T cells with subsequent inhibition of T-cell-mediated tumour cell apoptosis. This results in HCC immunotolerance and a permissive environment for tumour growth. PD-1, programmed death ligand; B7-H1 human B7 homolog 1; ECM, extracellular matrix

Three monoclonal antibodies against PD-1, and one against B7-H1 have been developed and promising Phase 1 data has been reported [149]. In one study, varying degrees of tumour regression were found in colon, renal and lung cancers and melanoma and a significant increase in tumour lymphocyte infiltration was noted [150]. This has been extended to a second clinical trial where responses were seen in 16 out of 39 patients with advanced melanoma [151]. These early clinical studies further demonstrated encouraging safety data. In the context of HCC, a Phase 1, dose escalation study investigating the effects of anti-PD-1 therapy is currently underway in patients with advanced HCC (NCT01658878), however results have yet to be reported. Some of the immunomodulatory effects of HSC in HCC are summarised in Fig. 2.

### Therapeutic approaches to targeting HSC and HSC signalling

HSC represent a small percentage of cells within the liver, and specific therapeutic targeting of HSC remains challenging. Recently, transgenic mice have been developed that allow reliable fluorescent labeling or genetic manipulation in HSC and myofibroblasts [152, 153]. These transgenic mice will hopefully prove useful not only in elucidating the molecular mechanisms in HSC that regulate the stroma-HCC interface, but also in facilitating the identification of rational, new therapeutic targets in hepatocarcinogenesis.

If a targetable, HSC-dependent pathway driving hepatocarcinogenesis is identified, cell-specific therapy is conceivable, albeit not entirely straightforward. ECM homeostasis is a key physiological process and modifying HSC functions may impair this, with potential for severe adverse effects. Practically, delivering drugs to HSC is hindered by a lack of multiple transport receptors and endocytic capacity. Furthermore, candidate compounds may include siRNA and cytokines, which have a short half-life in plasma following systemic administration, hindering therapeutic efficacy [154].

To overcome these problems, a number of groups have explored active targeting of HSC to deliver therapeutic compounds. This involves coupling the selected compound to a carrier possessing a specific receptor-binding ligand, or an antibody.

Carriers recently employed have included an antibody to the synaptophysin receptor on HSC, and a liposome specific to the vitamin A receptor on HSC [155, 156]. Furthermore Poelstra *et al.* have used proteins substituted with a sugar moiety that binds the mannose-6-phosphate-IGFII receptor [157]. They have also utilised a peptide that binds the PDGF receptor-β, [158] to deliver a protein or an adenovirus to HSC [159, 160]. An RGD-peptide which binds to RGD-binding integrins has also been used to create a carrier that accumulates in HSC [161, 162]. Of note, the carrier molecules used must fit strict criteria such as low immunogenicity, and high stability, biocompatibility and selectivity, if they are to translate into clinical practice. Moreover, the target receptors on HSC should be selectively expressed and ideally upregulated during disease activity. A further challenge is the requirement for endocytosis of the construct following target receptor binding. This can be particularly problematic in the case of biological therapeutics, which usually fail to withstand the endosomal degradation process.

**Fig. 3:**
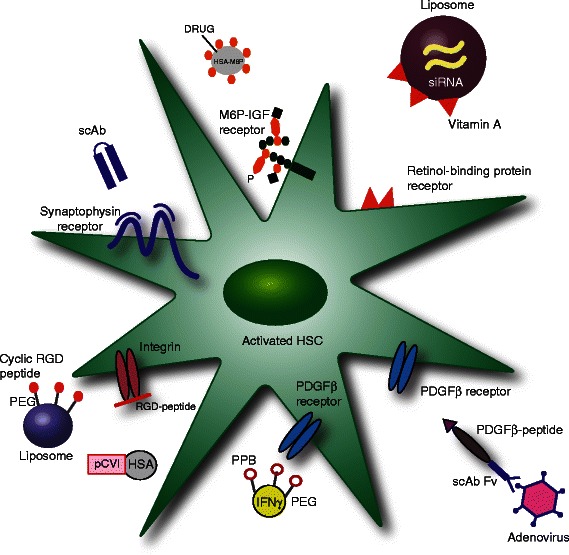
Therapeutic approaches to targeting HSC. HSC have been targeted by coupling a compound to a carrier possessing either a HSC-specific receptor-binding ligand or an antibody. Carriers utilised include: a monoclonal human single chain antibody (scAb) fragment to synaptophysin [155]; a sugar moiety that binds the mannose-6-phosphate (M6P) insulin-like growth factor receptor [157]; a liposome specific to the vitamin A (retinol-binding protein) receptor [156]; PDGFβ-peptide [160]; PDGFβ receptor recognising peptide (PPB) [164]; an RGD peptide bound to a liposome or coupled to human serum albumin (HSA) [159, 162] scAb Fv, single chain antibody variable fragment; PEG, polyethylene glycol; pCVI, 10 cyclic peptide moieties that recognise collagen type VI receptors

With these challenges in mind, Bansal *et al.* subsequently developed a recombinant protein construct to deliver interferon gamma (IFNγ) to HSC [163]. This elegant system transported the signalling moiety of IFNγ to the PDGF-receptor with a carrier molecule that was simplified and miniaturised. They found that IFNγ could be effectively delivered to human HSC *in vitro*, and to mouse HSC *in vivo*. Furthermore, the targeted fusion proteins were shown to ameliorate hepatic fibrosis in CCl_4_-treated mice [163–165]. This suggests that directing a cytokine to HSC is a feasible and potentially tractable therapeutic approach, both in the context of developing new treatments for patients with liver fibrosis, as well as HCC. Therapeutic approaches to targeting HSC are summarised in Fig. 3.

## Conclusions

Treatment options for HCC are still severely limited. Recently, increasing evidence has suggested that HSC are key regulators of hepatocarcinogenesis, likely through a variety of mechanisms, including direct effects on malignant hepatocytes, and indirectly via modulation of the peri-tumoural stroma and immune response. Further elucidation of the molecular mechanisms underpinning the crosstalk between the HSC, stromal and tumoural compartments will hopefully allow multi-faceted and personalized treatment of HCC in the future. For example, agents with a direct anti-tumoural effect could be combined with therapies that inhibit HSC-mediated angiogenesis and fibrogenesis. What has become increasingly clear is that neither the tumour nor the microenvironment can be viewed in isolation, rather that successful HCC therapies will need to be directed at counteracting the synergistic components of this complex relationship.

## Abbreviations

B7-H1: human B7 homolog 1
CAF: Cancer-associated fibroblasts
CCl_4_: Carbon tetrachloride
DEN: Diethylnitrosamine
ECM: Extracellular matrix
EGF: Epidermal growth factor
EMT: Epithelial-mesenchymal-transition
FAK: Focal adhesion kinase
FGF: Fibroblast growth factor
HCC: Hepatocellular carcinoma
HGF: Hepatocyte growth factor
HSC: Hepatic stellate cells
IFNγ: Interferon gamma
IGF: Insulin-like growth factor
iHSC: Intratumoural HSC
IL: Interleukin
MMPs: Matrix metalloproteinases
MDSC: Myeloid-derived suppressor cells
PAMPs: Pathogen-associated molecular patterns
PI3K: Phosphatidylinositol 3-kinase
PDGF: Platelet-derived growth factor
PDL-1: Programmed death ligand 1
TLRs: Toll-like receptors
TGF-β: Transforming growth factor-beta
TIMP-1: Tissue inhibitors of metalloproteinases 1
Tregs: Regulatory T-cells
VEGF: Vascular endothelial growth factor
